# Turkey’s transition to face-to-face schooling during the COVID-19 pandemic

**DOI:** 10.55730/1300-0144.5343

**Published:** 2022-04-02

**Authors:** Mahmut ÖZER, H. Eren SUNA, Matjaz PERC, Sadri ŞENSOY, Sevil UYGUN İLİKHAN

**Affiliations:** 1 Ministry of National Education, Ankara, Turkey; 2 Faculty of Natural Sciences and Mathematics, University of Maribor, Maribor, Slovenia; 3 Complexity Science Hub Vienna, Vienna, Austria; 4 Department of Medical Research, China Medical University Hospital, China Medical University, Taichung, Taiwan

**Keywords:** School reopening, face-to-face education, COVID-19, educational equality, vaccination

## Abstract

**Background/aim:**

The COVID-19 pandemic majorly disrupted conventional schooling and many countries maintained educational services through distance education. The duration of school closures in Turkey was longer than most OECD countries, thus Turkey prioritized school reopenings in the 2021–2022 academic year to mitigate possible negative outcomes of closures. Here we study the compatibility of implications for school reopenings in Turkey with these practices and assess the first semester of face-to-face schooling.

**Materials and methods:**

We have used document analysis to present and compare the practices in Turkey with international practices. We also used a comparative approach to assess the coherence between policies in Turkey and international suggestions.

**Results:**

We find that vaccination rates of teachers and education staff are quite high in Turkey. Other practices, mandatory face masks, class-based closures and quarantine policies, are also in agreement with international practices. These steps are supported with frequent cleaning and ventilation of school environments, as well as with social distancing measures in schools.

**Conclusion:**

Consequently, the rate of daily closed classrooms has been kept below 1%, and the patterns of closures and openings are in general agreement with the changes of positive cases in the Turkish society. The net rate of closed classrooms decreased with the decline of quarantine days in Turkey. We hope that these insights will inform about school openings and contribute to best practices for face-to-face schooling.

## 1. Introduction

The new type of coronavirus (COVID-19) has had major social and economic impacts around the world since its emergence in late 2019 [1,2]. Many countries began taking measures against spread of the virus in early 2020, when COVID-19’s effects and rate of spread were not yet clearly known. The first measures, such as frequent handwashing and personal hygiene, social distancing, and the face mask usage in indoor environments and other personal protective equipment (PPE), were not enough to prevent the virus and the COVID-19 pandemic has later defined as a “global pandemic” by the World Health Organization (WHO) [3]. After this declaration, protective measures tightened all over the world and expanded to include curfews and restrictions in closed areas [4].

The restrictions imposed due to the pandemic have had profound effects on social dynamics [5,6]. Education is one of the areas significantly impacted by these negative effects [7,8] because schools, which are among the main sources of social and cultural mobility, have been closed in many countries within the scope of pandemic measures. Thus, for the first time in known history, more than 94% of the world’s student population was simultaneously affected by school closures, with numbers reaching up to 99% in low-income countries [8]. This means that 1.6 billion students were removed from face-to-face education activities in schools.

With the closure of schools, many countries quickly transitioned to distance learning to attempt to resume educational activities [9]. Although distance learning can provide continuity of education in the short term, the countries’ readiness to provide this remote instruction, coupled with the difficulties accessing the services provided, caused significant frustration and debate [9]. Technological difficulties that hinder access to distance education and digital literacy differences among students continue to perpetuate socioeconomic disparities in learning, both locally and globally [10,11]. The closure of schools also limits both peer and student-teacher interaction, which are critical for the development of many cognitive and social skills [12]. Moreover, the modes of delivery for distance education, as well as its quality and students’ access to these services vary greatly both be-tween countries and between regions within any given country [13,14]. Therefore, students are not equally affected by the learning losses caused by the pandemic, but rather experience its effects at distinct levels according to differences in opportunity [7]. Previous research on education in times of disaster has also indicated that when schools cannot provide face-to-face education, the resulting negative repercussions are difficult to compensate for and disproportionately affect disadvantaged students [13].

Consequently, studies conducted during pandemic-related school closures have found many negative impacts stemming from disrupted instruction [15]. These studies provide evidence of deepening inequalities and increased learning losses [16–18], weakening social skills [19], unhealthy diets [20], and a spike in psychological problems for students, as well as loss of employment for their families [21]. Therefore, countries faced with a difficult dilemma. On the one hand, the closure of schools is a necessary precaution to prevent the spread of the pandemic, but on the other hand, this measure can create deep and potentially irreparable divides across a society [22].

Moreover, schools face increasing pressure to reopen, thanks to the rapid distribution of vaccines and mounting research evidence that the virus poses a lower threat to children than adults [22–26]. In addition, modelling studies have shown school closures have an extremely limited effect on reducing the spread of COVID-19 [27]; thus, schools are instead encouraged to reduce class sizes, maintain social distancing, and encourage hygiene measures [28]. International organizations such as UNESCO, the OECD, the United Nations, and the World Bank have also begun advising countries to resume face-to-face education as soon as possible [22,29–31]. Heeding this advice, many countries have accelerated the vaccination of education personnel, implemented new hygienic measures at schools, and prioritized face-to-face education [32].

Since many countries have reopened schools and transitioned back to face-to-face education, local and international agencies have closely followed the spread of the pandemic and the effects of the preventative measures taken [33–36]. Studies have shown that the spread of pandemic in schools is extremely limited if education personnel and students wear masks and receive vaccinations, and if their educational environments are well ventilated and frequently cleaned [33–35,37]. Some studies have even indicated that, if the necessary precautions are taken, the transition to face-to-face education does not have a significant effect on increasing the social spread of the pandemic—thus, a causal relationship cannot be established in this context [36]. Therefore, the effect of opening schools on the spread of the pandemic can be extremely limited if the appropriate precautions are taken in a timely and consistent manner [38].

In this context, Turkey began experiencing the effects of the pandemic later than other European countries, with its first official case seen on March 11, 2020. The virus spread rapidly throughout the country, causing the closure of schools for the second semester of the 2019–2020 academic year and almost all of the 2020–2021 academic year. As a result, the Education Information Network (EBA) digital platform and EBA TV channels became the primary means of education in Turkey during this time [39]. After 1.5 years of distance education at all levels, Turkey became the OECD country with the second-longest span of COVID-19 related school closures, after Mexico [40].

After a year and a half away from school, Turkish students needed to return to face-to-face education, and the necessary measures were taken to start the 2021–2022 academic year as close to normal as possible. The Turkish Ministry of National Education (MoNE) considered examples from around the world and the policy documents of international institutions when creating the roadmap to return to face-to-face education, as well as the precautions for keeping students and school personnel safe. This study aims to evaluate the compatibility of the measures taken to reopen Turkish schools to face-to-face education on September 6, 2021 with international recommendations and to assess the first semester of face-to-face education. In line with this general purpose, we comparatively examine the measures taken for the transition to face-to-face education, the education process, and the changes in the number of COVID-19 cases across Turkey.

### 1.1. International policies to maintain face-to-face education

#### 1.1.1. Encouraging vaccination

Vaccination is considered the most effective tool to prevent COVID-19 and mitigate the spread of the pandemic, as is the case in many viral outbreaks [41,42]. Individuals who are vaccinated with a full dose have a very low probability of severe disease, as well as a relatively low death rate [43]. Turkey also initiated the COVID-19 Turkey Platform to promote the development of vaccination and drugs against COVID-19 pandemic [44]. Current vaccines also provide significant protection against new variants, especially the highly transmissible Delta variant that emerged in mid-2021 [41]. Thus, many countries have prioritized vaccinating teaching staff who have frequent contact with students. For example, the CDC has promoted initiatives and events to facilitate access to the vaccine, as well as encouraging vaccination of students (when age-appropriate) and their families, as well as education personnel [41].

#### 1.1.2. Follow-up on cases and quarantine practices

The CDC and international organizations emphasise that continuous monitoring of COVID-19 cases in schools is critical to closely track the course of the pandemic and make necessary interventions. These monitoring tools are particularly important to ensure that education authorities intervene in schools with positive cases and protect the health of students and teachers. Identifying cases quickly and quarantining the infected individuals away from schools are both critical to maintaining face-to-face education. Thus, experts recommend establishing a monitoring system in cooperation between education and health authorities to ensure continuous follow-up.

Many countries employ rapid quarantining of cases and close contacts to further mitigate the spread of disease in schools. To ensure the continuity of face-to-face education, many countries—including France, Belgium, and the Netherlands—quarantine students at the class level, rather than quarantining the entire school. This practice prevents students who study at the same school but do not have close contact with positive cases from being deprived of face-to-face education.

After vaccination gained momentum globally, the CDC revised its guidance on quarantining; stating that fully vaccinated students and teachers can continue face-to-face schooling with a negative PCR test, even if they are in close contact. Based on the results of a new study on the spread of vaccination and the effects of the virus, schools have widely adopted quarantine periods shorter than 14 days. The CDC stated that a shorter quarantine of 7–10 days can be appropriate if close monitoring tests yield negative results [45].

#### 1.1.3. Use of face masks in indoor areas

Beyond vaccination, one of the prominent measures to reduce the spread of the pandemic is the use of face masks. The CDC [45] recommends that students and education personnel use face masks in schools, especially indoors; this guidance extends to all individuals over the age of 2, regardless of vaccination status. Since masking is one of the most important precautions together with vaccination, it is recommended that students use face masks both in school environments and during transportation to school.

The use of face masks is still important for preventing the spread of the pandemic, even when teachers and students are vaccinated. A recent study by [46] indicated that the probability of catching COVID-19 is 3.5 times higher in schools without face masks than in schools where masks are worn. In the state of Texas, schools saw significant increases in COVID-19 cases when the state’s governor banned mask mandates [47]. One study of Texas schools found that the number of positive cases was 96% higher for students and 61% higher for education personnel in schools where masking was optional [47]. Although children are less vulnerable to COVID-19 than adults, they still risk transmitting the virus to others at home and elsewhere if they do not wear face masks at school.

#### 1.1.4. Cleaning and ventilation of schools

School common areas, where students often assemble and come into close contact with one another, pose a significant risk for the spread of the virus. In this context, experts recommend adequate ventilation and frequent cleaning of common areas in schools [45,48].

#### 1.1.5. Maintaining social distancing

Social distancing between students is essential for conducting safe face-to-face education in schools during the COVID-19 pandemic. The CDC recommends maintaining approximately 2 m (6 feet) between students both inside and outside of the classroom. However, the CDC later revised its guidance to approximately 1 m (3 feet) if students wear face masks and take hygienic precautions [45]. To keep the social interaction at the lowest level, the CDC, OECD, and UNESCO advice reducing the number of students in the same environment and grouping them together. Such grouping can reduce the interaction between students during school entry and exit, passing time between classes, and lunch breaks.

## 2. Methods

We designed the presented study through a descriptive approach with a comparative perspective. Descriptive studies focus on describing the phenomenon in details as they are in nature [49]. Based on the fact that our aim is to describe the practices and outcomes of school reopenings in Turkey, we compared these practices and their outcomes from other countries within a descriptive manner.

We used the “COVID-19 e-tracking system” of MoNE to collect official data about number of closed classrooms and vaccination rates of teachers. The MoNE tracking system is integrated with Ministry of Health and it provides live data on these variables. The data of daily positive COVID-19 cases in Turkey are collected via official statistics of Ministry of Health. Teacher vaccination rate data from diverse countries is collected through UNESCO, survey outputs of National Education Association and American Federation of Teachers, and related official government announcements.

We used document analysis method to analyse the outputs of policies in Turkey and the coherence of these policies with international recommendations. The document analysis is a qualitative method for systematic review of documentary evidence to answer the research questions [50]. We consider the policy recommendations for safety school reopenings and sustaining face-to-face education of CDC [8,31,41,45,51] and OECD [30,48] to evaluate the policies of Turkey. In this manner, we grouped the common recommended policies in these materials, explain their importance and contribution to sustaining face-to-face education and discuss the coherence of practices in Turkey with these recommendations.

## 3. Findings

We begin by considering suggested practices for the transition to face-to-face education and measures taken in Turkey to that effect. The United States Centers for Disease Control and Prevention (CDC), UNICEF, the United Nations, and the World Bank have offered various guidance, informed by scientific data, for opening schools to face-to-face education in a healthy way during the COVID-19 pandemic [8,31,45,51]. These recommendations include promoting vaccination, using masks indoors, maintaining social distance, ensuring adequate ventilation, and applying general screening tests. This section outlines these international guidelines, before examining the steps taken in Turkey when switching to face-to-face education.

### 3.1. Encouraging vaccination

**Figure 1:**
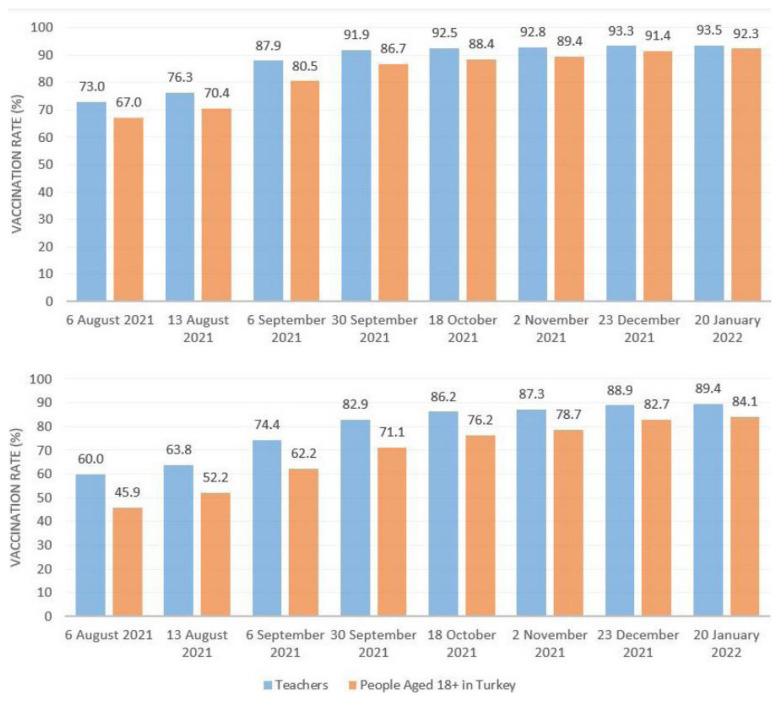
Vaccination rates of teachers between 6 August 2021 and 20 January 2022.^*^ a. One dose vaccination rates. b. Two doses vaccination rates. *The vaccination rates are collected through COVID-19 e-Tracking System of Ministry of National Education.

The most critical step taken to open schools for face-to-face education in Turkey involved increasing the vaccination rates of education personnel in contact with students. For this purpose, teachers were included among the priority groups for vaccination, to encourage them to get vaccinated as soon as possible. In addition, unvaccinated teachers were subject to compulsory PCR tests every two weeks. These practices rapidly increased the teacher vaccination rate nationwide; Figure 1 illustrates this change in the vaccination rates of teachers over time and compares teachers’ vaccination rates with the rates for all individuals in Turkey aged 18+.

As evident in Figure 1, teacher vaccination rates in Turkey were relatively higher than those of the general population even before September 6, when schools were opened, and continued to increase with the start of the school year. Over five months, approximately 21% more teachers have received vaccine doses. During this period, the vaccination rates of teachers continued to be higher than the overall vaccination rates in Turkey. As of January 20, 2022, the proportion of education personnel who received at least one vaccine dose has reached approximately 94%. In addition, nearly 5% of teachers have been infected with COVID-19 and developed antibodies, bringing the total percentage of teachers in Turkey protected from COVID-19 to approximately 99%.

**Figure 2:**
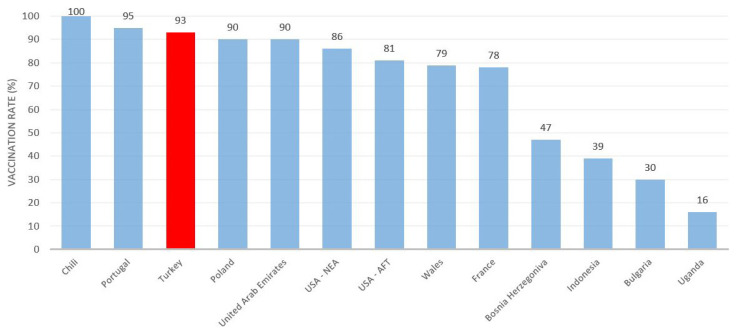
COVID-19 vaccination rates of teachers in various countries.^*^ *The data used were obtained as follows: USA (National Education Association-NEA and American Federation of Teachers-AFT, 17 June 2021), Bosnia Herzegovina (UNESCO, 10 October 2021), Bulgaria (UNESCO, 10 October 2021), France (McNicoll, 2 September 2021), Wales (Chrysanthos, 30 September 2021), Poland (Marek, 11 March 2021), Portugal (UNESCO, 10 October 2021), Chili (UNESCO, 10 October 2021), Uganda (UNESCO, 10 October 2021).

Figure 2 compares the vaccination rates of teachers in Turkey with those of teachers in other countries. It can be observed that the vaccination rate for teachers in Turkey are higher than those of their counterparts in most other countries. While the vaccination rate for teachers in Turkey is similar to those of Portugal and Poland, the rate is higher than those in the United States, France, Bulgaria, Bosnia and Herzegovina, Wales, and the United Arab Emirates. In an education system with nearly 1.2 million teachers, increasing the vaccination rate to this level is one of the most important steps taken for a healthy transition to face-to-face education.

**Figure 3:**
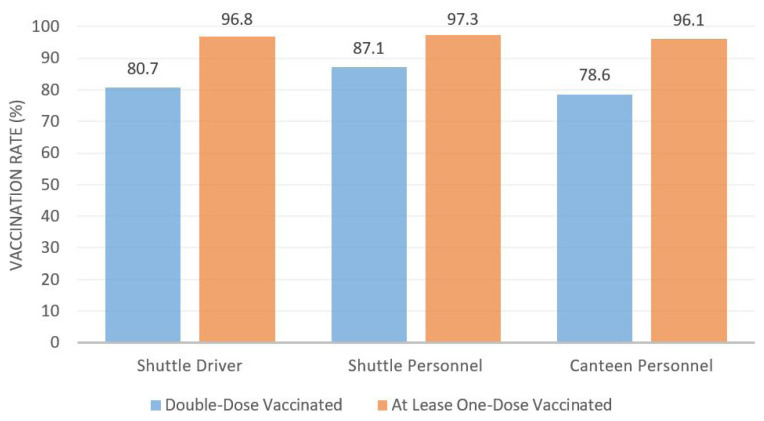
Vaccination rates of support personnel.^*^ *The vaccination rates are collected through COVID-19 e-Tracking System of Ministry of National Education.

In addition, these high vaccination rates were not limited to teachers, but rather extended to all personnel who have contact with students. Moreover, bus drivers, service personnel, and cafeteria workers are also required to wear masks. Vaccination rates of support personnel in Turkey are given in Figure 3.

As Figure 3 demonstrates, the vaccination rates of nonteaching school staff are also very high. Thus, the vast majority of personnel that students can come into contact with are vaccinated, from their teachers, to the support staff who supervise their transport, breaks, and meals.

### 3.2. Follow-up on cases and quarantine practices

The Turkish Ministry of Health and MoNE created the “MoNE COVID-19 e-Tracking System” before face-to-face schooling resumed nationwide on September 6, 2021. This system synchronously follows positive cases at the provincial, district, and school levels, and the data provided form the basis for all quarantine decisions. To ensure the continuity of face-to-face education and alleviate the effects of the pandemic on-site, quarantine is applied at the class level in the event of a positive case. Originally, the quarantine period across Turkey was 14 days, but this was reduced to 10 days as of October 3. At the end of the quarantine period, students in the affected classes return to face-to-face education. While quarantined, students receive distance education-based live lessons to ensure the continuity of their education. In addition, the MoNE provides tablets and/or computers to students in need during this process.

### 3.3. Use of face masks in indoor areas

During the transition to face-to-face education in schools in Turkey, the use of face masks was made compulsory for all students and education personnel, both in school and on the shuttles used to transport students to schools. While students and education personnel were expected to come to school with face masks, face masks were also provided to all schools for use when needed. Hygiene kits, including face masks and disinfectants, were distributed to education personnel by the MoNE every month from the beginning of the term.

### 3.4. Cleaning and ventilation of schools

In Turkey, the budget allocated for school cleaning needs for the 2021–2022 academic year was significantly higher than ever before—nearly 10 times its typical size. This measure was designed to ensure that schools had access the cleaning materials they would need throughout the semester. During this process, 45,000 new janitorial staffs were employed to work in schools. The Ministry of Health and the Science Board collaborated to create a “Guide for the Precautions to be taken in Schools during the COVID-19 Pandemic”, which outlined requirements for daily cleaning of all schools, alongside frequent ventilation of indoor environments. In schools with central ventilation systems, these systems were maintained and arranged according to the requirements of the pandemic period.

### 3.5. Maintaining social distancing

Differences in school environments and contexts, coupled with shifting numbers of students make it difficult to make one-size-fits-all decisions for schools across Turkey, as in many other countries. Based on this necessity for flexible options, schools were given the authority to monitor the process and make decisions together with their provincial administrations. Thus, in coordination with provincial administrations, schools were able to change class times, as well as recess and entry/exit times to maintain social distancing. In addition, if school capacity made it difficult to implement pandemic measures, schools could confer with their provincial administration to switch to dual education.

### 3.6. Transition to face-to-face education and the spread of the pandemic in Turkey

**Figure 4:**
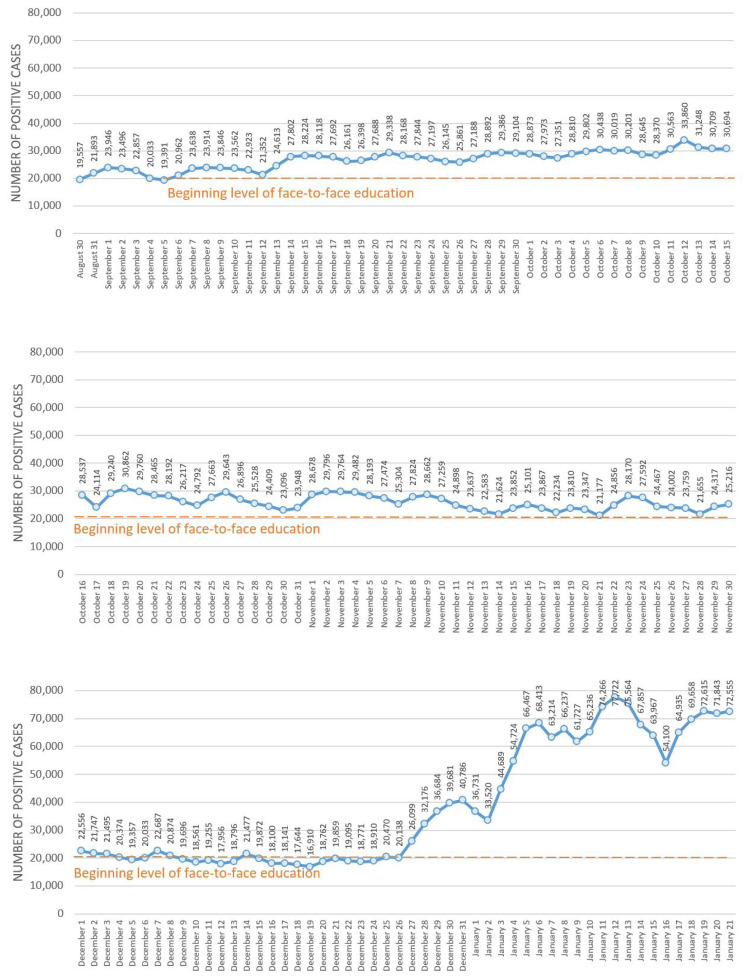
Number of COVID-19 cases in Turkey before and during the first semester of face-to-face education in schools.

The measures detailed above enabled Turkey to complete the first semester of face-to-face education in schools across the country. Throughout this process, monitoring the return of the country’s approximately 18 million students and 1.2 million teachers to their schools has been critical to chart the continued course of the pandemic and compare the process with that of similar countries. Figure 4 charts the number of COVID-19 cases in Turkey both before and after schools reopened.

As seen in Figure 4, after the face-to-face opening of schools on September 6, Turkey saw a partial increase in the number of cases nationwide, before exhibiting a downward trend again at the end of the first month. In addition, fluctuations in increments of cases were also observed in the week before face-to-face education.

After the first three months, the number of positive cases decreased to the level of September 6, which face-to-face education has started. Therefore, it is reasonable to assume that the increase in cases during the initial weeks of school reopening in Turkey is related to increased movement before the start of term, as well as the transition to face-to-face education. In the middle of December, the number of positive cases even decrease to a lower level than the school reopenings. However, in late December when the Omicron variant officially observed in Turkey the daily positive cases increased remarkably similar to the neighbouring countries.

**Figure 5:**
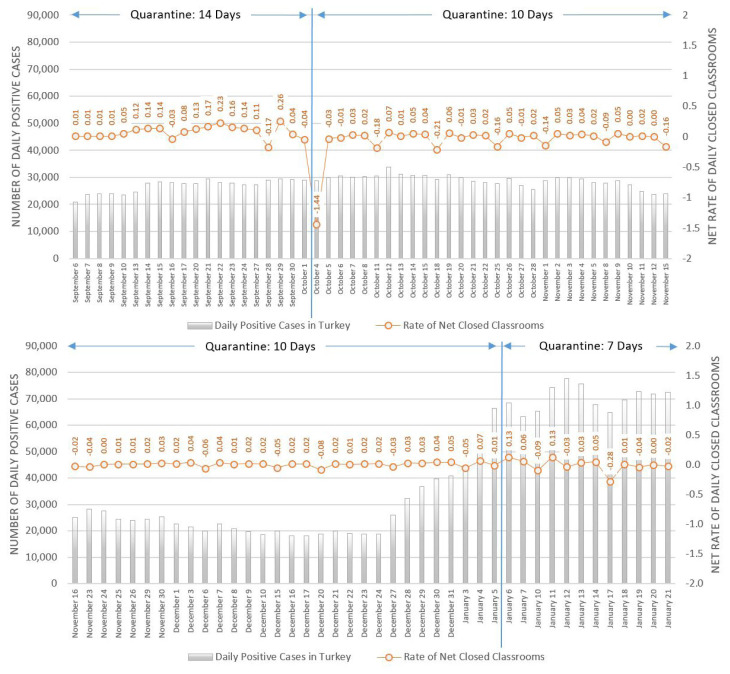
Percentage of closed classes and case numbers in Turkey after the transition to face-to-face schooling.^*,**^ *The daily positive COVID-19 cases data is collected through official statistics of Ministry of Health and rate of daily closed classrooms is gathered via COVID-19 e-Tracking System of Ministry of National Education. **Negative rates of daily closed classrooms occur when the rates of opened classrooms after quarantine are higher than rates of closed classrooms.

Figure 5 illustrates the net rate of closed classes due to positive cases and the change in the number of positive cases across Turkey during the semester of face-to-face education in schools. The net percentage of classes closed due to the pandemic was well below 1% throughout this period. The graph demonstrates that the net daily closed class rates in Turkey were closely related to the change in the number of cases in adults over age of 18. It is also important to note that, the net rate of closed classrooms in late December and January did not change remarkably despite the dramatic increase in positive cases in Turkey. In other words, the change in daily case numbers and net closed class rates exhibited a similar pattern with a lag. This finding serves as a potential indicator that the number of cases in schools is also affected by cases in the community—thus, cases in schools can be fed by nonschool sources.

Another important finding seen in Figure 5 is that reducing the quarantine period from 14 days to 10 days resulted in a significant decrease in closed class rates. After the decision to decrease the quarantine period, the number of days in which the number of opened classes exceeded the number of quarantined classes also increased. Similarly, the decrease of quarantine period to 7 days in January also helped to keep the net rate of closed classrooms at low levels. Thus, since September 6, when schools in Turkey switched to face-to-face education, the already low percentage of closed classrooms decreased even more after the quarantine period was reduced to 10 days, kept at similar levels with 7 days of quarantine and showed similar changes to the general number of cases in Turkey.

**Figure 6:**
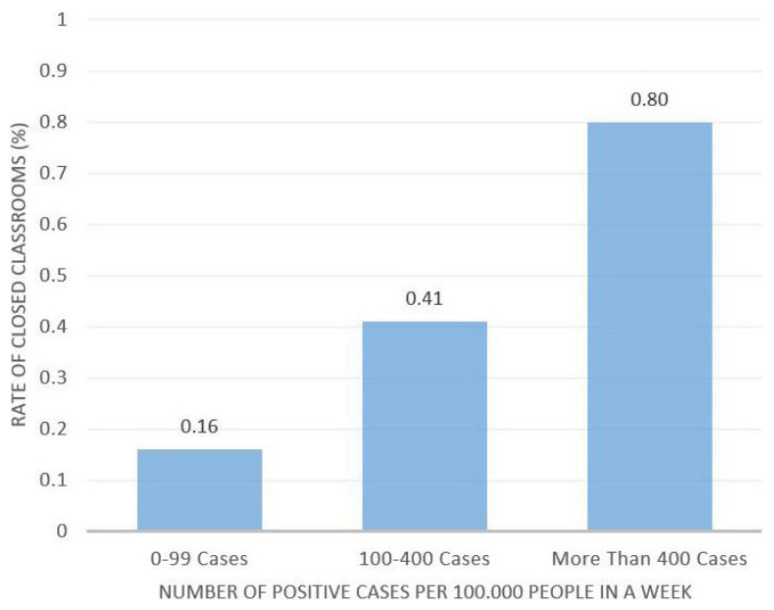
Closed class rates in provinces in different groups by number of positive cases per week.* *The daily positive COVID-19 cases data is collected through official statistics of Ministry of Health and rate of daily closed classrooms is gathered via COVID-19 e-Tracking System of Ministry of National Education.

To better visualise the relationship between the number of cases nationwide and the rates of closed classes, the provinces were grouped according to their number of cases and the change in their rates of closed classes. The results of this analysis are given in Figure 6.

As illustrated, there is a significant relationship between the number of positive cases per week and the percentage of closed classes. In this context, the rate of closed classes in provinces with more than 400 positive cases per hundred thousand people is approximately 2 times that of provinces with 100–400 cases, and approximately 4 times higher than in provinces with 0–99 cases.

## 4. Discussion and Conclusion

Turkey, like many other countries, closed schools in March 2020 to prevent the spread of the COVID-19 pandemic and quickly moved classes to distance education platforms [52]. However, the fact that the school closure period in Turkey was longer than most OECD countries [40], coupled with the scale of the education system has deepened the effects of school closures nationwide. The MoNE thus planned to resume face-to-face education in schools during the 2021–2022 academic year and took the necessary precautions to prepare schools, staff, and students before the semester began. This study discussed the appropriateness of the measures taken for the transition to face-to-face education in Turkey and aimed to evaluate the current situation following the first semester of in-person instruction.

As evidenced in the previous sections, the MoNE took important steps to ensure that face-to-face education resumed in a safe and sustainable manner. These steps included encouraging the vaccination of all education personnel in contact with students, requiring mask wearing in schools, allocating the necessary funds to supply masks and disinfectants to schools, establishing a pandemic monitoring system integrated with the Ministry of Health to closely monitor infections, and employing new personnel to clean schools every day. Providing schools with the opportunity to group students, partially rearrange class hours, and switch to dual education in coordination with provincial administrations has also increased their ability to implement local measures against the effects of the pandemic. To ensure the effective implementation of these practices, the MoNE appointed a deputy principal and psychological counsellor at each school to carry out and monitor COVID-19 prevention measures. One week before the start of the term, all teachers were given training on the transition to face-to-face education during the pandemic period. Additional informational activities have been carried out for various education stakeholders, who also have access to official lines of communication and informational resources.

These steps taken by the MoNE safeguarded the transition of students to face-to-face education at school. The findings obtained during this first semester study mirror the experiences of many other countries that have opened their schools for face-to-face education. The partial increase in the number of cases following the transition to face-to-face education is consistent with the data from other countries such as Wales, the United States, and England [35]. The increases in these first weeks can be attributed to mobility during the summer period, the return of parents to work, and increasing social connections, as well as the opening of schools [53]. The number of cases, which increased partially when classes resumed on September 6, were in decline again as of the second month of school and reached to beginning level at the third month. Additionally, despite the dramatic increase in number of positive cases with Omicron variant in late December in Turkey, the net rate of closed classrooms kept well below 1%. In addition, the similarity in the patterns of closed classes and the number of cases in the general population, coupled with the higher rates of closed cases in provinces that face greater rates of COVID-19 infection, indicates that the cases in schools may actually be caused by community spread, rather than transmission within school environments. In other words, findings related diverse closed classroom rates in regions with different positive cases indicated that the spread of cases in the community is also reflected in schools.

As of the end of first semester, the number of quarantined classes were still at low level, as positive cases showed an increasing trend across Turkey and the quarantine period was reduced to 7 days. Therefore, taking steps in accordance with international practices made a significant contribution to the first semester of successful continuation of face-to-face education in Turkey. Considering that there are approximately 18 million students and approximately 1.2 million teachers in the Turkish education system, the importance of these findings speaks for itself.

To increase the sustainability of in-person schooling, the MoNE has also started to produce the types of rapid antigen tests that have been applied in many other countries. Vocational education and training (VET) institutions which have played an important role in meeting Turkey’s economic and technological needs since the first days of the pandemic [54–57] have undertaken the production of these rapid tests. With the introduction of such measures, mass testing will soon be possible for young students who cannot be vaccinated. Thus, early detection of asymptomatic cases will further limit the spread of COVID-19 and reduce the rate of closed classes. Therefore, the continued implementation of these multipronged pandemic safety measures and the introduction of rapid antigen tests will promote the sustainability of face-to-face education across Turkey.
